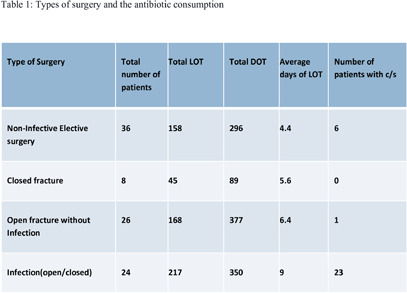# A Structured Thematic Audit Analysis with Consensus Building Leads to Optimized Antibiotic Use and Clinical Decision Pathways.

**DOI:** 10.1017/ash.2025.277

**Published:** 2025-09-24

**Authors:** Prasannakumar Palanikumar, Priscilla Rupali, Hanna Alexander

**Affiliations:** 1 Christian Medical College; 2 Christian Medical College Vellore

## Abstract

**Introduction:** Pre-authorization and prospective audit and feedback, though effective interventions for reducing antibiotic use, require manpower, time and can impinge on prescriber autonomy. We describe a unique approach to optimizing antibiotic use. **Methodology:** The antimicrobial stewardship program at our hospital is physician-led and supported by clinical pharmacists. To reduce time and manpower, we adopted a collaborative approach of structured audits. A baseline phase measured antibiotic consumption, mapped antibiotics to clinical syndromes, and documented inappropriate antibiotic use about choice, dose and duration. We then went to an intervention phase where for a month, prospective audit and feedback was performed for all the patients in the department in real time, communicated and discussed with a liaison from the treating team. At the end of this period, we presented data regarding antibiotic consumption and the proportion of justified antibiotic use in terms of choice, dose, and duration compared to the baseline phase. Literature evidence of appropriate antibiotic use was presented along with actionable data where gaps had been identified. **Results:** Structured thematic audits were conducted across seven key departments, including Medicine, Surgery, Orthopedics, Obstetrics and Gynecology, Urology, Hematology, and Emergency Medicine. As an example, the data on Orthopedics is presented here. The audit was done over one month across three general wards, and 94 patients were recruited. The antibiotic consumption was DOT/100PD=78.2, and the average length of therapy was 6.2 days. The antibiotic utilization for the broad infectious specific syndrome is shown in Table 1. Non-infective elective surgery and closed fracture received 4.4 and 5.6 mean days of antibiotics, which was deemed unnecessary. However, no institutional antibiotic protocol for open fractures (considered contaminated) existed. On discussion with the entire orthopedics department, a consensus was reached on antibiotics for open fractures with or without contamination for a maximum of 72 hours or until wound closure. Other areas where antibiotics could be optimized according to standard guidelines were also agreed upon and reinforced. This meeting resulted in consensus building and collaborative clinical decision pathways adopted into our institutional antibiotic guidelines. **Conclusion:** This unique thematic structured audit approach enhanced judicious antimicrobial prescribing practices, leading to consensus building across the hospital. It also led to changes in policy, fostering ownership and breaking the hierarchical model of stewardship, shifting accountability to the primary departments. It also reduced the time and resources required for the AMS team.

**Figure:**